# Evolutionary genomics of the pandemic 2009 H1N1 influenza viruses (pH1N 1v)

**DOI:** 10.1186/1743-422X-8-250

**Published:** 2011-05-21

**Authors:** Yanhua Qu, Ruiying Zhang, Peng Cui, Gang Song, Ziyuan Duan, Fumin Lei

**Affiliations:** 1 Key Laboratory of Zoological Systematics and Evolution, Institute of Zoology, Chinese Academy of Sciences, Beijing, 100101, China; 2 Institute of Genetics and Developmental Biology, Chinese Academy of Sciences, Beijing 100101, China; 3 Graduate School of the Chinese Academy of Sciences, Beijing, 100039, China

## Abstract

**Background:**

A new strain of human H1N1 influenza A viruses was broken out in the April 2009 and caused worldwide pandemic emergency. The present study is trying to estimate a temporal reassortment history of 2009 H1N1 viruses by phylogenetic analysis based on a total 394 sequences of H1N1viruses isolated from swine, human and avian.

**Results:**

Phylogenetic trees of eight gene segments showed that viruses sampled from human formed a well-supported clade, whereas swine and avian lineages were intermixed together. A new divergence swine sublineage containing gene segments of 2009 H1N1 viruses was characterized, which were closely related with swine viruses collected from USA and South Korea during 2004 to 2007 in six segments (PB2, PB1, PA, HA, NP and NS), and to swine viruses isolated from Thailand during 2004 to 2005 in NA and M. Substitution rates were varied drastically among eight segments and the average substitution rate was generally higher in 2009 H1N1 than in swine and human viruses (*F*_*2*__,23 _= 5.972, *P *< 0.01). Similarly, higher *d*_N_/*d*_S _substitution ratios were identified in 2009 H1N1 than in swine and human viruses except M2 gene (*F*_2, 25 _= 3.779, *P *< 0.05). The ages of 2009 H1N1 viruses were estimated around 0.1 to 0.5 year, while their common ancestors with closest related swine viruses existed between 9.3 and 17.37 years ago.

**Conclusion:**

Our results implied that at least four reassortments or transmissions probably occurred before 2009 H1N1 viruses. Initial reassortment arose in 1976 and avian-like Eurasian swine viruses emerged. The second transmission happened in Asia and North America between 1988 and 1992, and mostly influenced six segments (PB2, PB1, PA, HA, NP and NS). The third reassortment occurred between North American swine and avian viruses during 1998 to 2000, which involved PB2 and PA segments. Recent reassortments occurred among avian-to-swine reassortant, Eurasian and classical swine viruses during 2004 to 2005. South Korea, Thailand and USA, were identified as locations where reassortments most likely happened. The co-circulation of multiple swine sublineages and special lifestyle in Asia might have facilitated mixing of diverse influenza viruses, leading to generate a novel virus strain.

## Background

In April 2009, a new strain of human H1N1 influenza A viruses was identified in Mexico and USA, which caused a rapid global spread and moved the global pandemic alert level to phase 6 on 11 June 2009 [1]. This virus has been widely spread over 170 countries from April to June 2009 [1], and has the ability to persist in the human population, potentially with more severe clinical consequences [2]. Initial genetic characterization of the 2009 H1N1 viruses suggested swine as their probable source, on the basis of sequence similarity to previously reported swine influenza viruses [3-5]. The NA and M gene segments are in the Eurasian swine genetic lineage. The other six gene segments are well clustered into North American triple-reassortant swine lineage. More specially, the HA, NP and NS gene segments belong to classical swine lineage; while the PB2, PB1 and PA gene segments are derived from the North American swine, avian and human H3N2 reassortant lineage [4-6].

Swine has been believed to play a vital role in interspecies transmission of influenza viruses, since it harbors receptors to both avian and human influenza virus strains. Pigs have been considered as a possible "mixing vessel" in which genetic material can be exchanged, with the potential to result in novel progeny viruses to which humans are immunological naive and highly susceptible [7]. Sporadic cases of swine influenza in human, combined with seroepidemiological studies demonstrate increased risk of swine influenza in occupationally exposed workers, highlighting the crucial role that swine may play in the development of new strains of influenza viruses [8-10]. Notably, human infection with H1N1 swine influenza has been a nationally notifiable disease in United State since 2007 [11].

H1N1 influenza viruses were first isolated from swine in 1930 and have been shown to be antigenical highly similar to a recently reconstructed human 1918 A (H1N1) virus [12,13], and likely share a common ancestor [14,15]. From 1930 to late 1990s these classical swine influenza viruses circulated in swine and remained antigenically stable [4,16,17]. In 1998, a new reassortant swine virus, comprising genes from classical swine viruses and North American avian viruses, was reported as the cause of outbreaks in North American swine influenza, with subsequent establishment in pig populations. In Europe, avian H1N1 viruses were introduced to pigs and first detected in Belgium in 1979. This avian-like swine lineage emerged and subsequently prevailed in Asia after that time [18].

Given the evolutionary history of swine influenza viruses, it is likely that multiple reassortment events resulted in emergence of the novel 2009 human H1N1 viruses. However, the poor surveillance for swine influenza viruses fails to detect these potential reassortants, and where reassortment events most likely happened is also unclear. Considering the ongoing health risk posed by 2009 H1N1 viruses, it is very important to estimate evolutionary genomics of the novel pandemic viruses; to identify the potential reassortment events and where they probably occurred. We therefore undertook a detailed phylogenetic analysis in a total 394 sequences of H1N1 viruses taken from swine, human and avian from 1918 to 2009 to estimate a temporal reconstruction of reassortment history of 2009 H1N1 viruses. By employed a sophisticated Bayesian Markov Chain Monte Carlo (MCMC) approach, the time to the common ancestor and population dynamics of 2009 H1N1 viruses and their closest related swine viruses were also determined.

## Materials and methods

### Sequence data collection and alignment

H1N1 influenza viruses isolated from swine, human and avian hosts between 1918 and 2009 were studied. Their genomic sequences were accessed from NCBI before May 2009, and aligned using Mega 4.0 [19]. Eight genome segment alignment datasets (PB2, PB1, PA, HA, NP, NA, M and NS) were generated.

### Phylogenetic analyses

Phylogenetic trees of eight alignment datasets were reconstructed using the ML approach implemented in PyhML 2.4 [20], and with bootstrapping analyses of 1,000 pseudo-replicate datasets. The robustness of the ML tree topology was assessed by comparing the ML topology with the topologies sampled in the MCMC analyses performed in MrBayes version 3.1.2 [21,22]. The substitution model for each gene dataset was selected according to MrModeltest 2.2 [23]. Phylogenies were rooted with the H1N1 human virus in 1918.

### Estimating the rate of evolution and genetic diversity dynamics

Overall rates of evolutionary change (nucleotide substitutions per site per year) for swine, human, and 2009 human H1N1 viruses were estimated using the BEAST program 1.4.6 [24], utilizing the number and temporal distribution of genetic differences among viruses sampled at different times [25]. Estimate of evolution rate was not performed for avian viruses because limited sample size (n = 47). Previous analyses of human influenza A viruses evolution found that the uncorrelated exponential relaxed clock model provided a better fit to the H1N1 influenza viruses data [24], thus it was employed in estimating the average rates of nucleotide substitution.

To estimate the date of origin for 2009 H1N1 viruses, we preformed a Bayesian skyline plot (BSP) for each gene segment to estimate the time of the most recent common ancestor (TMRCA). TMRCA of 2009 H1N1 viruses and their closet related swine viruses was also estimated for comparisons. All estimates incorporated the General Time Reversible (GTR) and Γ4 model of DNA substitution as this model was consistently with the best supported one in MrModeltest 2.2. The uncorrelated exponential relaxed clock model was selected in estimations. The uncertainty in the estimates reflected in the 95% highest probability density (HPD), and in each case chain lengths were run for sufficient time to achieve convergence.

### Measurement of selection pressures

To determine the overall selection pressure faced by each gene segment in 2009 H1N1 viruses, we compared the *d*_N_/*d*_S _rates of this lineage with those of swine and human virus lineages. Swine and human viruses after 1976 were selected in this analysis because either widely sampling was available or the earliest origin of the 2009 H1N1 viruses could go back to avian-to-swine reassortantment in 1976. We estimated the mean numbers of nonsynonymous substitutions (*d*_N_) and synonymous substitutions (*d*_S_) per site using the single-likelihood ancestor counting (SLAC) method accessed through the Datamonkey interface (http://www.datamonkey.org). In all cases, *d*_N_/*d*_S _estimates were based on neighbour-Joining trees under the substitution model selected by website.

## Results

### Phylogeny of H1N1 influenza viruses and genetic origin of 2009 H1N1 human viruses

**Figure 1:**
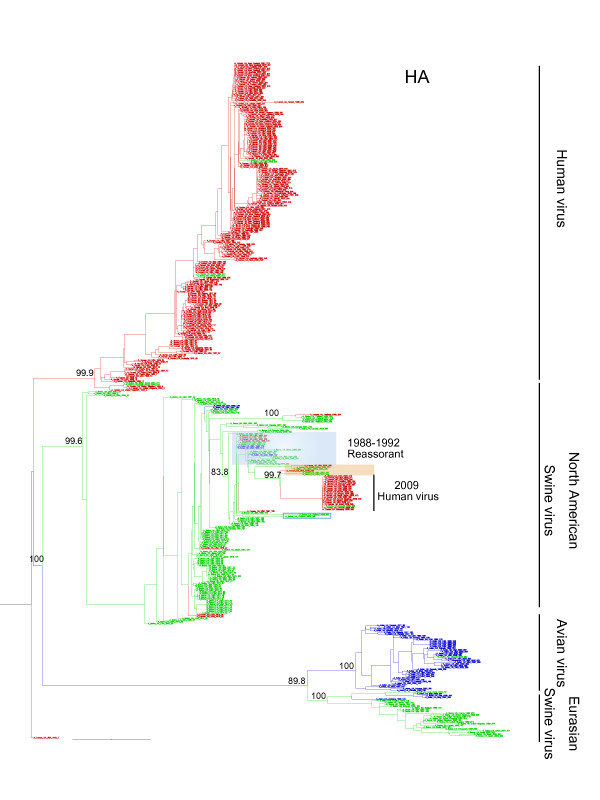
**Phylogenetic trees of H1N1 influenza viruses**. ML phylogenies reconstructed from (A) HA gene; (B) NA gene; (C) PA gene. Human lineage is showed in red; while swine and avian are characterized in green and blue, respectively. Only the topological supports for major lineage or sublineages and reassortant viruses are showed and summarized from 1,000 ML bootstrap replicates. Transmissions among swine, avian and human viruses between 1988 and 1992 are highlighted in blue. The closest related swine viruses of 2009 H1N1 viruses are highlighted in red. Phylogenetic trees for five other gene segments can be found in Additional file 1.

**Figure 2:**
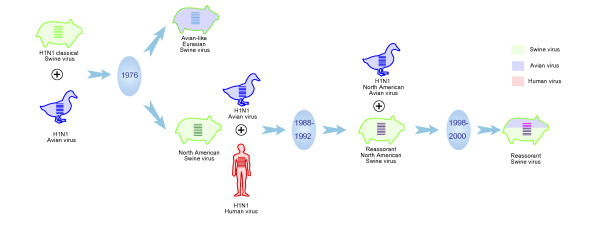
**Reconstructed reassortment events leading to the emergence of 2009 H1N1 influenza viruses**.

Phylogenetic trees of all eight segments demonstrated viruses sampled from human formed a well-supported clade in all phylogenies with the maximum likelihood support rate of 80 to 100%, whereas swine and avian lineages were intermixed together (Figure 1, Additional file 1). Viruses sampled from swine in North America and Asia tended to cluster together (classical swine sublineage), and formed a paraphyly topology with human clade. Swine viruses isolated from Europe and Asia after 1977 clustered with European avian viruses and nested into avian lineage (Eurasian swine sublineage). In PB2 and PA, some swine viruses were nested into North American avian clade, of which were mostly sampled after 1998 and isolated from North America and Asia (avian-to-swine reassortant sublineage). The PB1 had undergone more complex reassortments, which occurred among North American avian, human and swine viruses (triple reassortant swine sublineage) (Additional file 2). Notably, many swine, avian and human H1N1 viruses isolated from 1988 to 1992 intermixed together in all eight gene segments (Figure 1 and Figure 2). These mixed clades were often linked by a long branch with their relatives (Figure 1 and Additional file 1).

**Figure 3:**
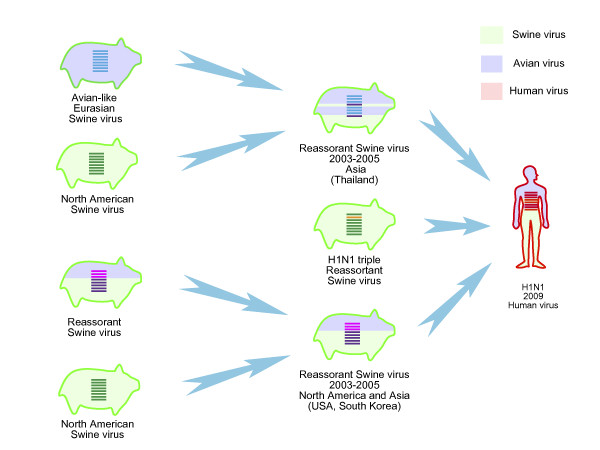
**Reassortment events involved with the genetic parents of the 2009 H1N1 influenza viruses**. Asia, particularly Thailand, was identified as the place where reassortments between Eurasian and classical swine sublineages occurred; alternatively, Asia and North America, especially as South Korea and USA, was where reassortments between classical and avian-to-swine reassortant sublineages happened.

Phylogenetic trees characterized a new divergence swine sublineage containing gene segments of 2009 H1N1 viruses, which were apparently mostly similar to classical swine sublineage in HA, NP and NS, to avian-like Eurasian swine sublineage in NA and M, to avian-to-swine reassortant swine sublineage in PB2 and PA, and to triple reassorant swine sublineage in PB1 (Figure 1 and Figure 2). Within new divergence swine sublineage, 2009 H1N1 viruses were most closely related with A/swine/USA/2005, A/swine/USA/2007, A/swine/South Korea/2004 and A/swine/South Korea/2005 in six gene segments (PB2, PB1, PA, HA, NP and NS). Comparisons of the nucleotide similarity showed 98 to 99% homology (Additional file 3). These ancestral relatives contained genes from both classical and avian-to-swine reassortant swine sublineages, indicating reassortments occurring in 2004 and 2005, particularly in North America and Asia (Figure 3). For NA and M, the closest related viruses were A/swine/Thailand/2005 and A/swine/Thailand/2004, and their similarity was around 97 to 98% (Additional file 4).

### Substitution rate estimations and population dynamics

**Figure 4:**
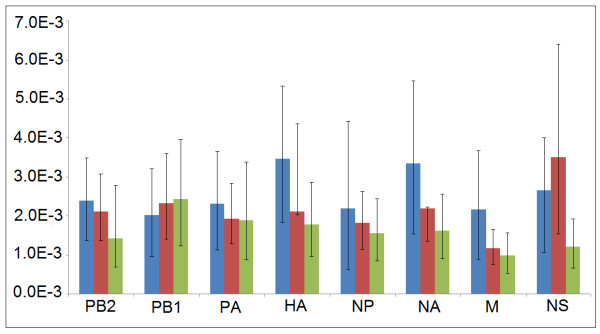
**Rates of nucleotide substitution in H1N1 influenza viruses**. Mean substitution rates are showed for each gene segment, with 95% lower and upper HPD values shown as error bars. Blue shows the 2009 H1N1 viruses, while red and green indicate swine and human viruses, respectively.

The substitution rates were varied among eight gene segments, ranging from 3.51* 10^-3 ^for NS to 1.16* 10^-3 ^for M in swine viruses; and from 2.88 * 10^-3 ^for PB1 to 0.99* 10^-3 ^for M in human viruses (Figure 4). The average substitution rate was generally higher in the 2009 H1N1 viruses than in swine and human viruses (*F*_*2*__,23 _= 5.972, *P *< 0.01). However, differences were more significant in HA, NP and NA and M (*F*_*2*__,11 _= 6.668, *P *< 0.05), but not in others (*F*_*2*__,11 _= 2.12, *P *= 0.176).

**Figure 5:**
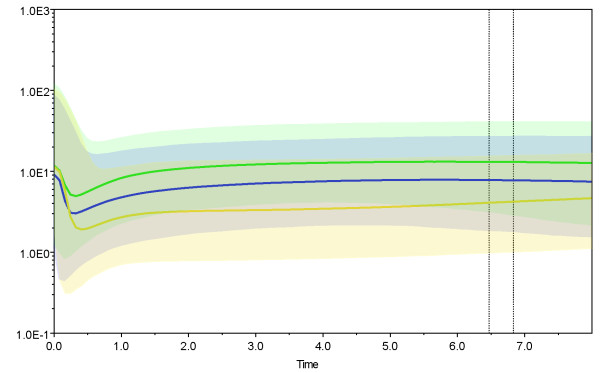
**Estimations population dynamics of 2009 H1N1 viruses and their closest related swine viruses**. Green, blue and yellow shows HA, NA and PA, respectively, which represented three different origins, especially as classical, Eurasian and avian-to-swine reassortant swine sublineages. Population dynamic estimates for classical, Eurasian and avian-to-swine reassortant swine sublineages can be found in Additional file 5. X-axis: time in years before present; Y-axis: estimated population size [units = Neτ, the product of effective population size and generation length in years (log-transformed)]. The median estimate and both 95% HPD limits are indicated.

The ages of the 2009 H1N1 viruses were estimated extremely recent (range 0.1 to 0.5 year). The common ancestors of 2009 H1N1 and closest related swine viruses existed between 9.3 and 17.37 years ago (Additional file 4, Table S1). BSP estimation showed an abrupt rise in population dynamic after a long-term constant population size in each gene segment (Figure 5 and Additional file 5).

### Diversifying selection in 2009, swine and human H1N1 virus lineages

**Figure 6:**
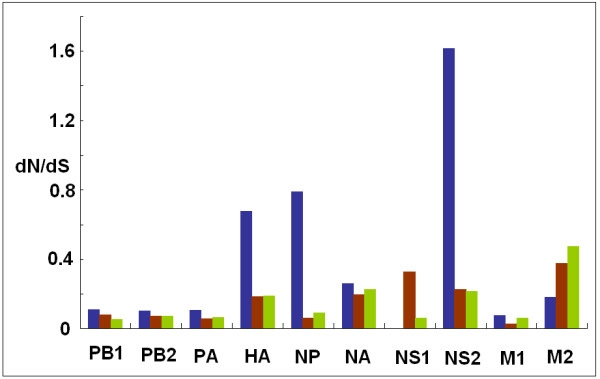
**Comparisons of *d***_**N**_**/*d***_**S **_**ratios among 2009 H1N1, swine and human viruses**. Blue showed 2009 H1N1 viruses; red and green indicated swine and human viruses, respectively. The *d*_N_/*d*_S _ratio estimate could not be performed for NS1 gene of 2009 H1N1 viruses because only few variable amino acid sites are available.

The higher *d*_N_/*d*_S _rates were observed in HA, NP and NS segments in 2009 H1N1 viruses (Figure 6). Similarly, HA and NS showed higher *d*_N_/*d*_S _values in swine and human virus lineages, in addition to NA and M (Figure 6). In general, 2009 H1N1 viruses showed a comparatively higher *d*_N_/*d*_S _substitution rate than those in swine and human viruses except in M2 gene (*F*_2, 25 _= 3.779, *P *< 0.05).

## Discussion

### Multiple reassortment events resulting in new pandemic 2009 H1N1 human viruses

Previous studies have classified new emerged 2009 H1N1 viruses a swine origin [4,5]. Sublineages of swine viruses in our phylogenetic trees belonged to different lineages of hosts, which suggest that multiple reassortment events occurred. It is thus important to recognize which reassortant groups are closely related to 2009 H1N1 viruses. These ancestral groups might give us clues of when and where these reassortant events have probably happened [4].

Phylogenetic analyses revealed that the initial reassortment event might occur in 1976, while gene segments of NA and M of 2009 H1N1 viruses derived from the Eurasian H1N1 swine sublineage. In 1976, an avian H1N1 virus strain was introduced to pigs and established a new avian-like H1N1 swine sublineage (H1N1 Eurasian swine) and circulated in European and Asian countries [4,5]. Between 1988 and 1992, swine, avian and human viruses frequently intermixed together, which mostly occurred in Asia and North America. These transmissions might influence PB2, PB1, PA, HA, NP and NS genes of 2009 H1N1 viruses. Furthermore, 2009 H1N1 viruses have undergone another reassortment between North American swine and avian viruses. This event occurred between 1998 and 2000, and gene segments of PB2 and PA derived from this avian-to-swine reassortant sublineage. The PB1 has experienced more complex reassortments, first of which occurred between North American avian and human viruses during 1978 to 1979. The new avian-to-human reassortant viruses underwent subsequently reassortments with North American swine viruses during 2000s. Investigations for the closest related ancestors of 2009 H1N1 viruses revealed recent reassortments occurred both between avian-to-swine reassortant and classical swine sublineages, and between classical and Eurasian swine sublineages. These results thus imply that 2009 H1N1 viruses might have acquired gene segments from different groups of parental H1N1 viruses, which underwent at least four reassortments and transmissions among swine sublineages or swine, avian and human lineages.

### Where reassortment events most likely happened

Detection of the closest ancestral genes for each of eight segments of 2009 H1N1 viruses may suggest where reassortment events most likely happened. Reassortments between classical and avian-to-swine reassortant swine sublineages occurred in South Korea and USA, while reassortments between Eurasian and classical swine sublineages were found in Thailand. These thus indicated North America and Asia might be the place where reassortments probably occurred. Classical and avian-to-swine reassortant swine sublineages have co-circulated in North America [26,27] and frequent reassortments between two sublineages have been detected and resulted in the generation of triple reassortant swine H1N1 and H1N2 viruses [4]. In Asia, the classical, Eurasian avian-like and avian-to-swine reassortant swine influenza viruses co-circulate, and extensive reassortments among three sublineages have been observed [5], with reassortments between Eurasian avian-like and reassortant swine sublineages occurring as early as 2003 (for example, Hong Kong). The co-circulation among multiple swine sublineages might have promoted opportunities of reassortments and tended to create the new influenza virus lineage.

Asia, especially Southeast Asia, is thought to be the epicenter for the influenza pandemics throughout history [28]. The special environment and lifestyle in Southeast Asia provide more chances for wild aquatic birds, domestic poultry, pigs and human to contact closely, and create the opportunities for interspecies transmission and generation of new reassortment influenza viruses.

### Population dynamics of 2009 H1N1 viruses and their closest related viruses

The common ancestors of 2009 H1N1 viruses and their closest related swine viruses might have been existed 9 to 17 years before; in contrast, the currently sampled 2009 H1N1 viruses shared a common ancestor around January 2009. It is possible that ancestors of 2009 H1N1 viruses have been cryptically circulating for several years before emergence in human. BSP analyses revealed a rapid growth in early 2001 after more continuous and relatively slow growth in a decade. In recent years, both classical and avian-to-swine reassortant swine influenza viruses have occasionally been isolated from humans [7,8,29], however, the lack of systematic swine virus surveillance allowed for undetected persistence and evolution of this potentially pandemic strain for many years. Thus systematic monitor and surveillance of H1N1 swine viruses for these regions, particularly Asian countries, should be required for future controlling of the outbreaks of new strain influenza viruses.

### Different evolutionary backgrounds in 2009 H1N1, human and swine influenza viruses

2009 H1N1 viruses showed comparatively higher substitution rates than those of human and swine viruses. Similarly, relatively high ratios of non-synonymous to synonymous substitutions per site (*d*_N_/*d*_S_) were also observed for all segments of 2009 H1N1 viruses except M2 gene. The difference was more significant in HA, NS and NP genes, most likely reflecting different selection pressures at eight gene segments [30].

The difference among evolutionary rates of swine, human and 2009 viruses might reflect various evolutionary backgrounds. Human and swine H1N1 viruses were characterized by low rates of evolutionary change, particularly at amino acid-changing sites, thus proposed to have reached an 'evolutionary stasis' [31]. These observations demonstrate that selective pressure is less intense, so that there is little selective requirement to repeatedly fix amino acid changes that evade host's immune responses [32]. However, a high evolutionary rate in the newly emerged 2009 H1N1 viruses might be due to adaptation to the new host species, indicating strong selective advantages. Alternatively, it could also be a general feature of intensively sampled emerging epidemics, which should be considered in the other evolutionary analyses such as the surveillance of the 2009 H1N1 viruses breakout.

## Conclusions

Considered ongoing health risk of 2009 H1N1 influenza viruses, it is necessary to detect potential reassortments involved in emergence of the novel viruses through the phylogenetic analyses. Results in the present study imply that at least four reassortments or transmissions probably occurred before H1N1 broke out. South Korea, Thailand and USA, were identified as locations where reassortments most likely happened. The co-circulation of multiple swine sublineages and special lifestyle in Asia might have facilitated mixing of diverse swine, avian and human viruses, leading to generate novel influenza viruses. 2009 H1N1 viruses showed comparatively higher selection than swine and human viruses in all gene segments except for M2. Our results also revealed that ancestors of 2009 H1N1 viruses existed between 9 and 17 years ago, suggesting that this virus strain has been circulating undetected for decade years and the importance of systematic surveillance of influenza viruses in swine, especially in Asian areas.

## Competing interests

The authors declare that they have no competing interests.

## Authors' contributions

This study was elaborated and launched by FML and ZYD, who leading two research groups on ornithology and genetics. YHQ and RY Z accomplished the data analyses and YHQ narrated the paper. PC did much effort on data collection and editing. GS participated in the work of manuscript preparation and revision. All authors have read and approved the final manuscript.

## Supplementary Material

Additional file 1 **Figure S1. Phylogenetic trees of H1N1 influenza viruses**. ML phylogenies based on PB1, PB2, NP, MP and NS gene segments respectively. Human lineage is showed in red; while swine and avian are characterized in green and blue, respectively. Transmissions among swine, avian and human viruses between 1988 and 1992 are highlighted in blue. The closest related swine viruses of 2009 H1N1 viruses are highlighted in red. Click here for file

Additional file 2 **Figure S2. Possible reassortments of PB1 in the emergence of 2009 H1N1 viruses**. The PB1 has undergone more complex reassortments, which occurred among North American avian, human and swine viruses. Click here for file

Additional file 3 **Table S1. Nucleotide identities (uncorrected *P *distance) between 2009 H1N1 viruses and their closest related swine viruses**. Click here for file

Additional file 4 **Table S2. Time of recent common ancestors (TMRCA) for 2009 H1N1 viruses and their closest related swine viruses**. Click here for file

Additional file 5 **Figure S3. Population dynamic estimates for classical, Eurasian and avian-to-swine reassortant swine sublineages**. X-axis: time in years before present; Y-axis: estimated population size [units = Neτ, the product of effective population size and generation length in years (log-transformed)]. The median estimate and both 95% HPD limits are indicated. Click here for file
