# Supplementary material for: Evolutionary genomics of the pandemic 2009 H1N1 influenza viruses (pH1N 1v)
Source: Virol J. 2011 May 21;8:250. doi: 10.1186/1743-422X-8-250 (PMC3201028; doi:10.1186/1743-422X-8-250)
Supplement: Additional file 1 — Figure S1. Phylogenetic trees of H1N1 influenza viruses. ML phylogenies based on PB1, PB2, NP, MP and NS gene segments respectively. Human lineage is showed in red; while swine and avian are characterized in green and blue, respectively. Transmissions among swine, avian and human viruses between 1988 and 1992 are highlighted in blue. The closest related swine viruses of 2009 H1N1 viruses are highlighted in red. [file 1743-422X-8-250-S1.PDF]

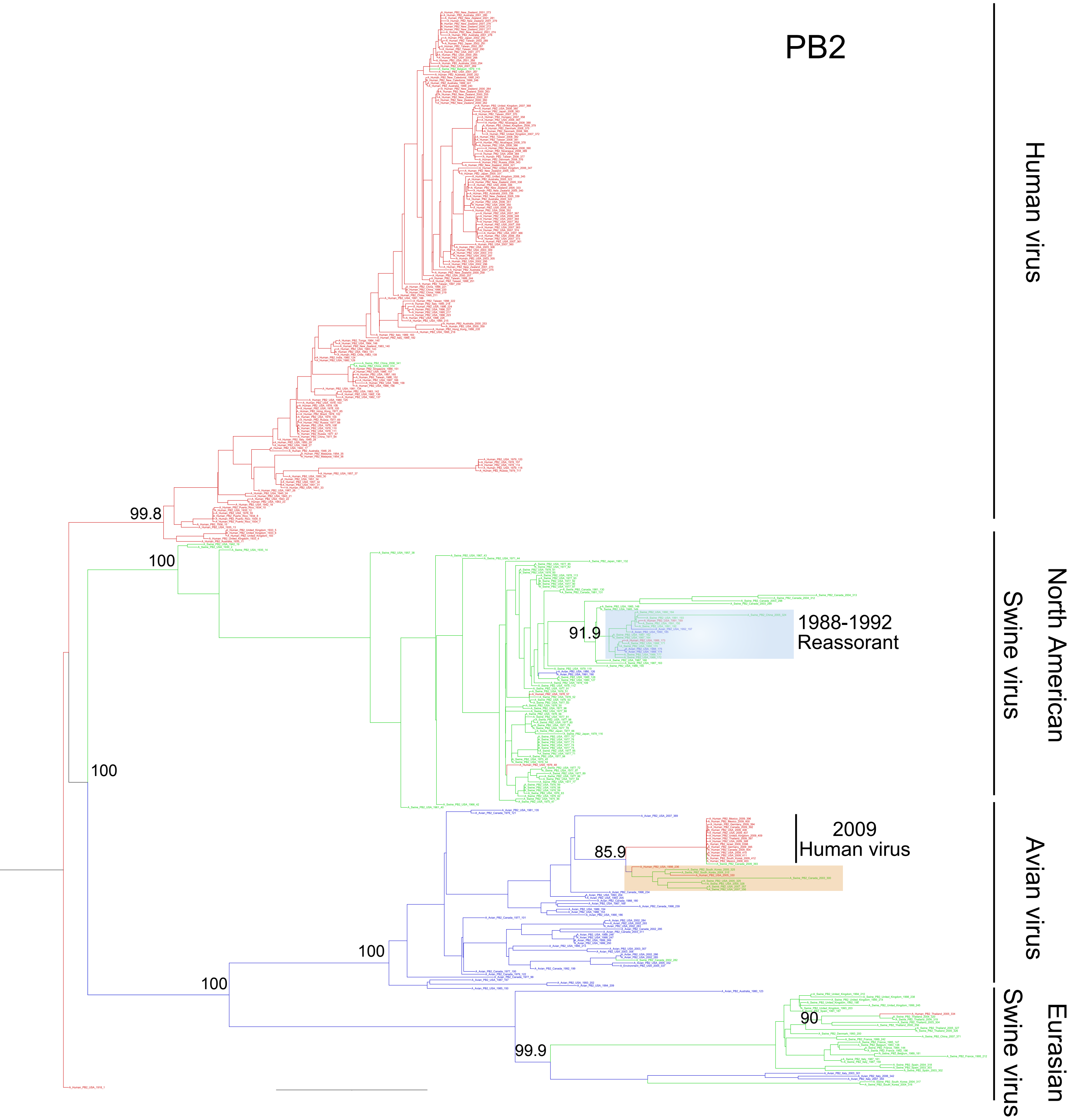
PB2
Human virus
North American Swine virus
Avian virus
Eurasian Swine virus
1988-1992 Reassorant
2009 Human virus
99.8
100
100
91.9
85.9
100
100
99.9
90

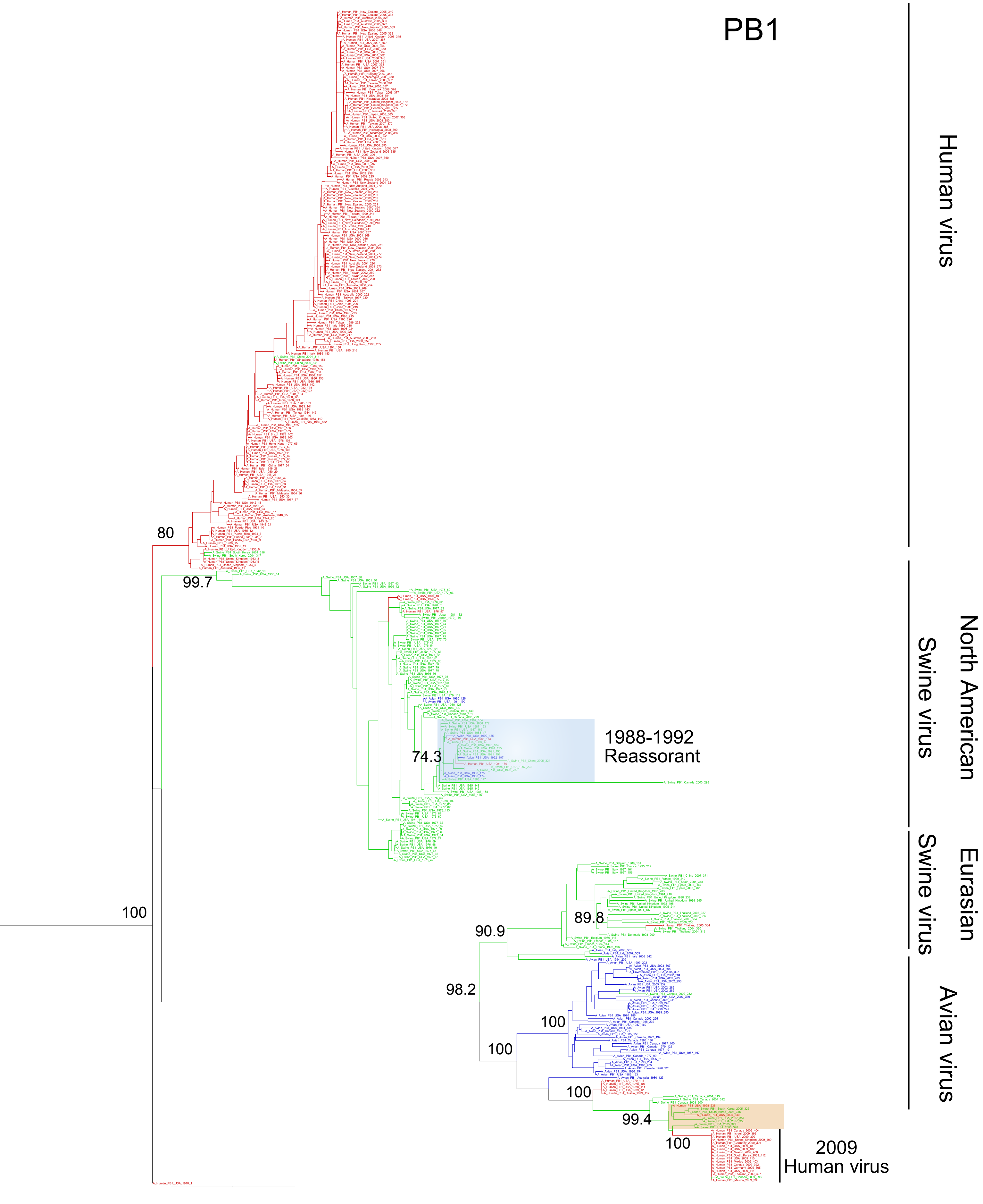

PB1
Human virus
North American
Swine virus
Eurasian
Swine virus
Avian virus
1988-1992
Reassorant
2009
Human virus
80
99.7
100
74.3
90.9
89.8
98.2
100
100
100
99.4
100

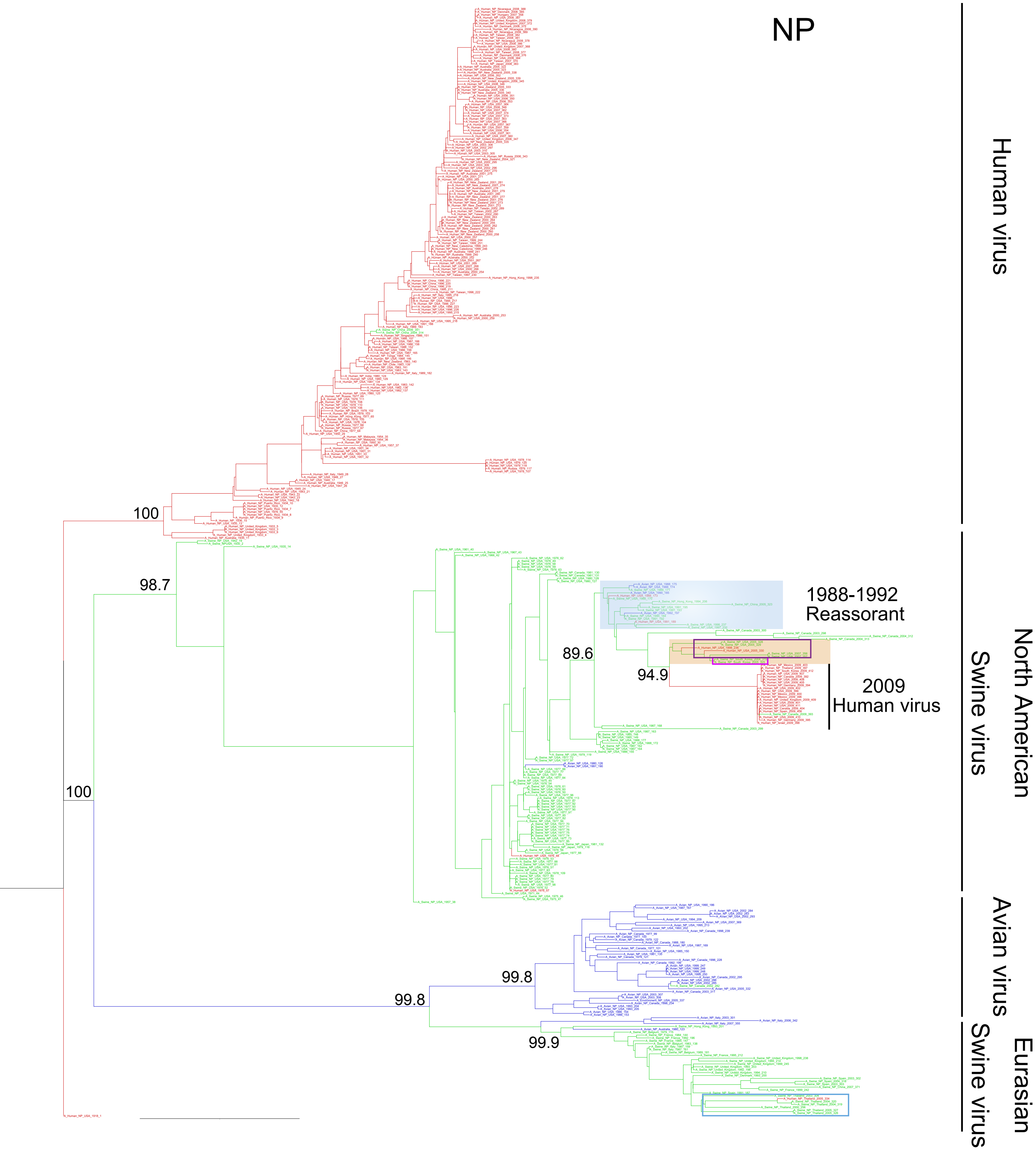

NP
Human virus
100
98.7
100
1988-1992
Reassorant
89.6
94.9
2009
Human virus
North American
Swine virus
99.8
99.8
99.9
Avian virus
Eurasian
Swine virus

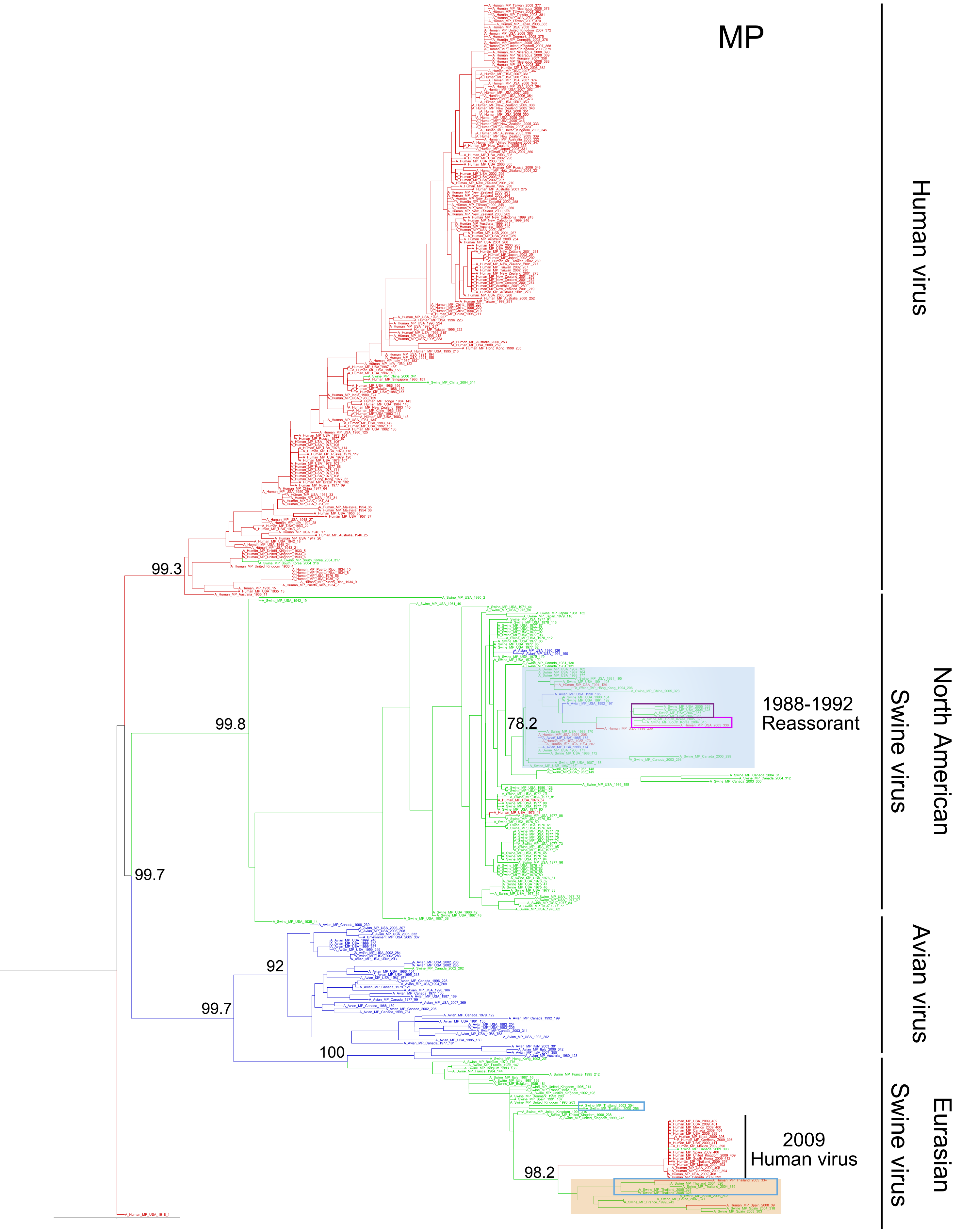
MP
Human virus
North American Swine virus
1988-1992 Reassorant
Avian virus
Eurasian Swine virus
2009 Human virus
99.3
99.8
99.7
78.2
92
99.7
100
98.2

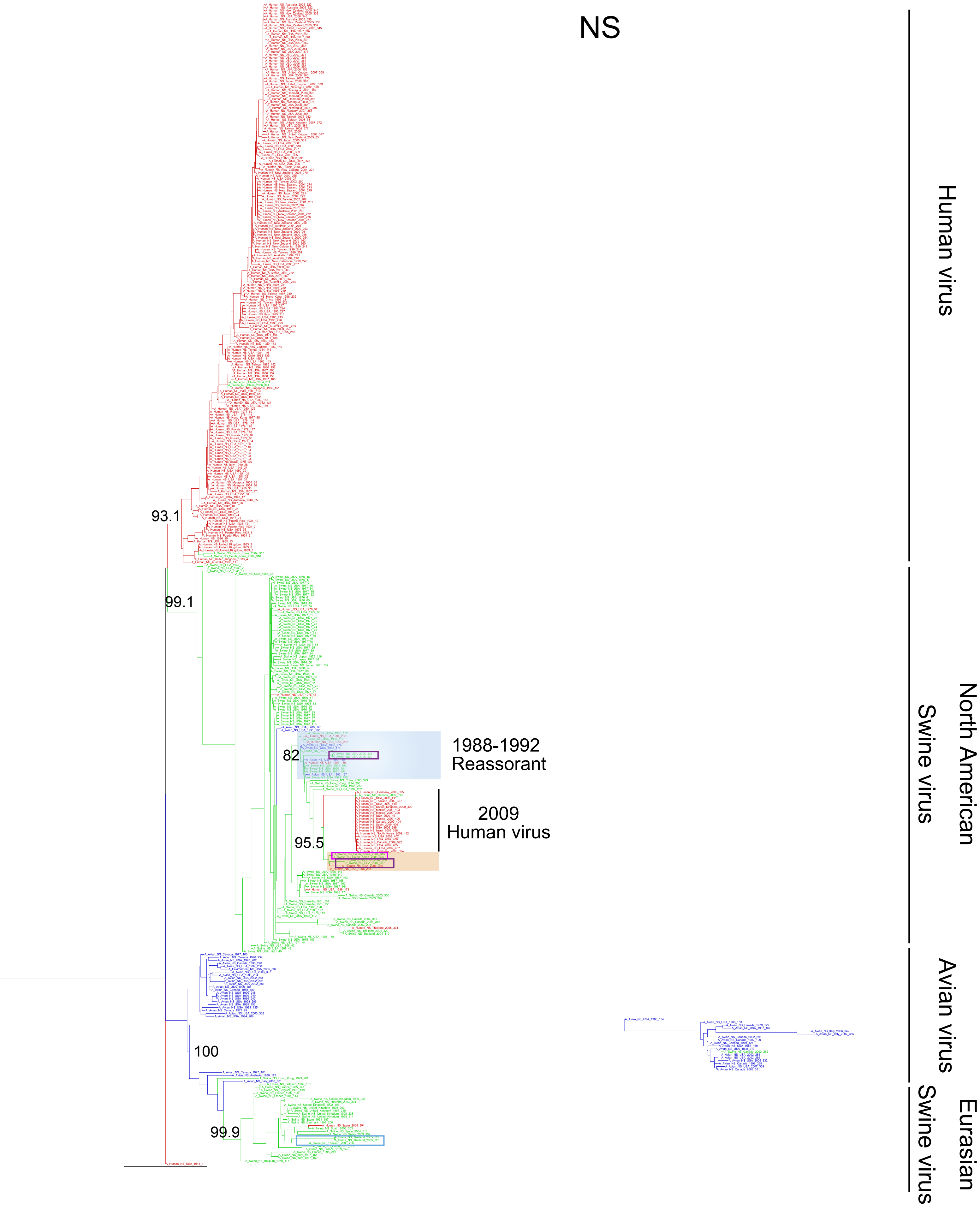
NS
Human virus
93.1
99.1
North American Swine virus
82
1988-1992 Reassorant
2009 Human virus
95.5
Avian virus
100
99.9
Eurasian Swine virus
