# Supplementary figures and images for: Evolutionary genomics of the pandemic 2009 H1N1 influenza viruses (pH1N 1v)
Source: Virol J. 2011 May 21;8:250. doi: 10.1186/1743-422X-8-250 (PMC3201028; doi:10.1186/1743-422X-8-250)

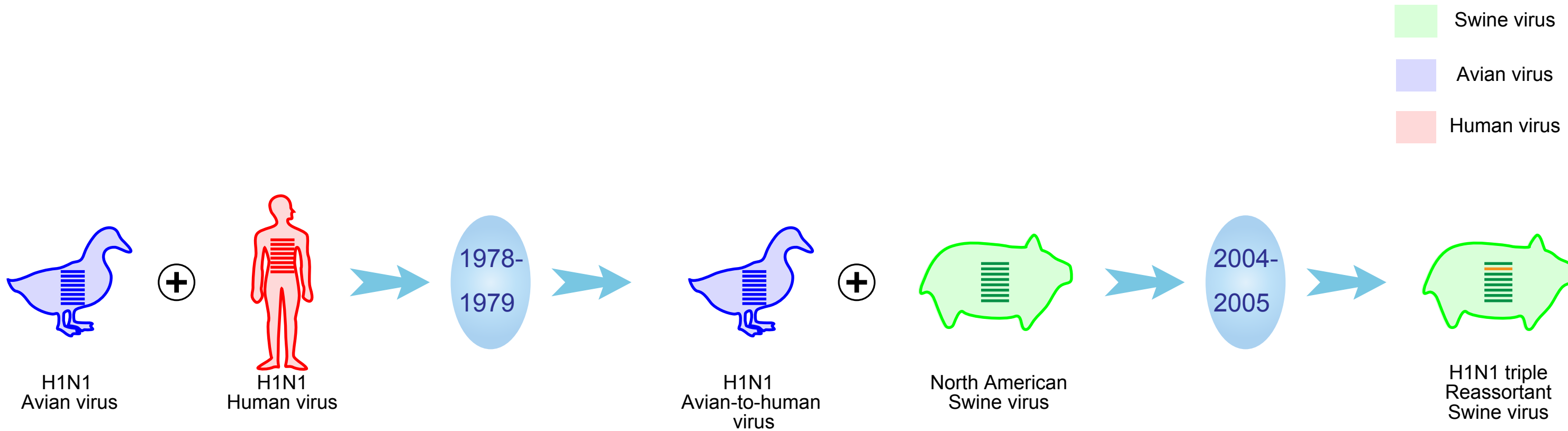

Supplement: Additional file 2 — Figure S2. Possible reassortments of PB1 in the emergence of 2009 H1N1 viruses. The PB1 has undergone more complex reassortments, which occurred among North American avian, human and swine viruses. [file 1743-422X-8-250-S2.PDF]

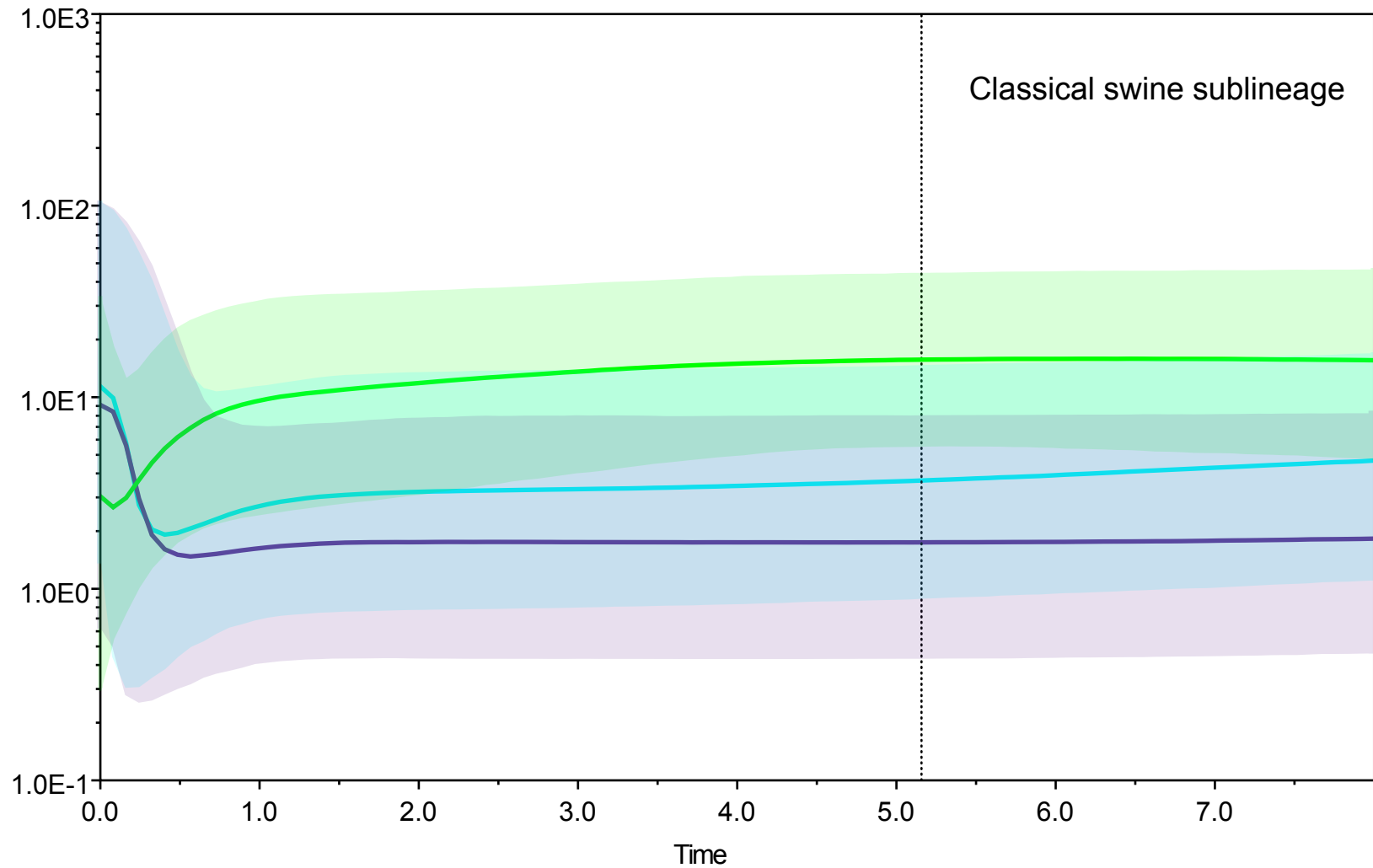

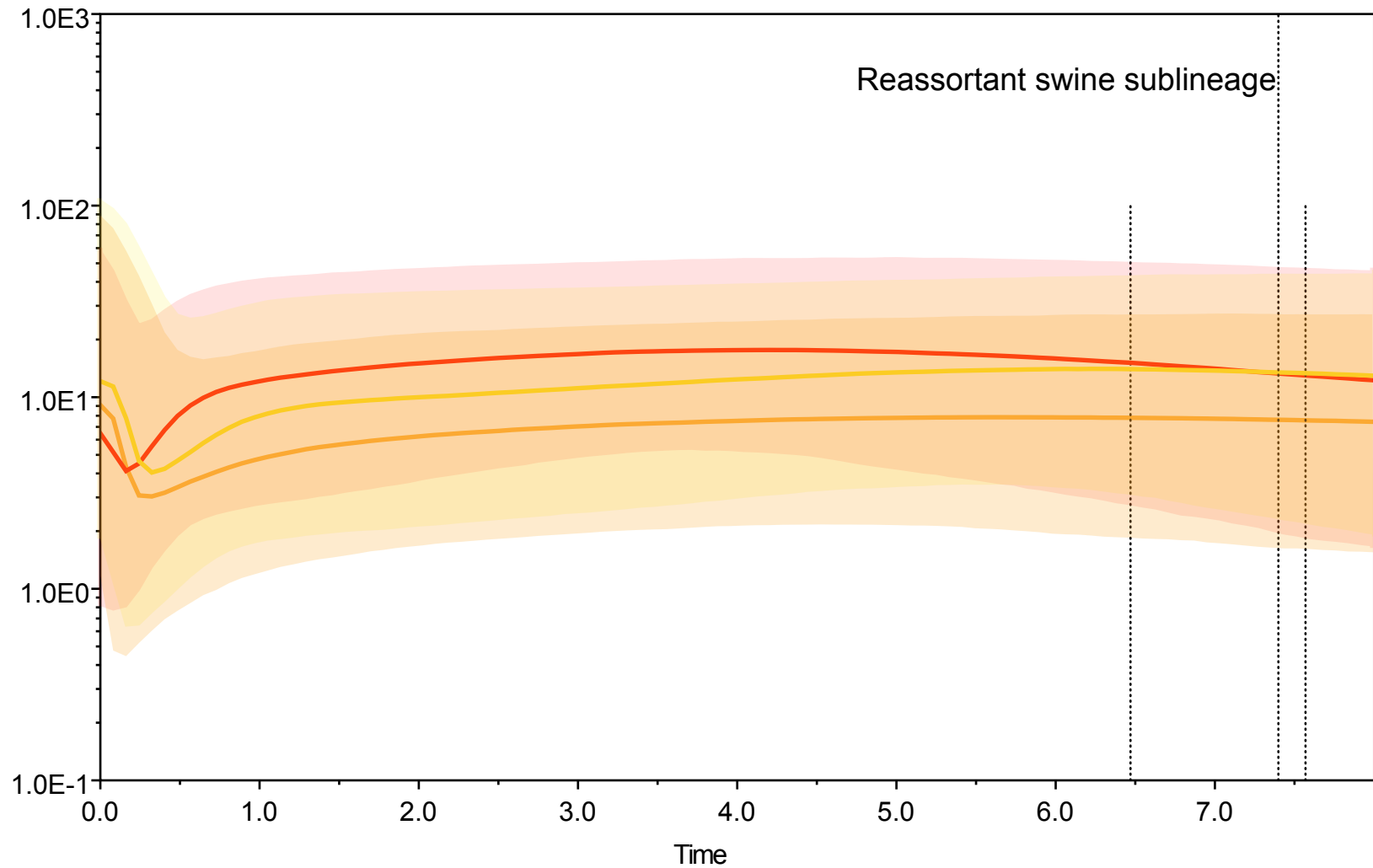

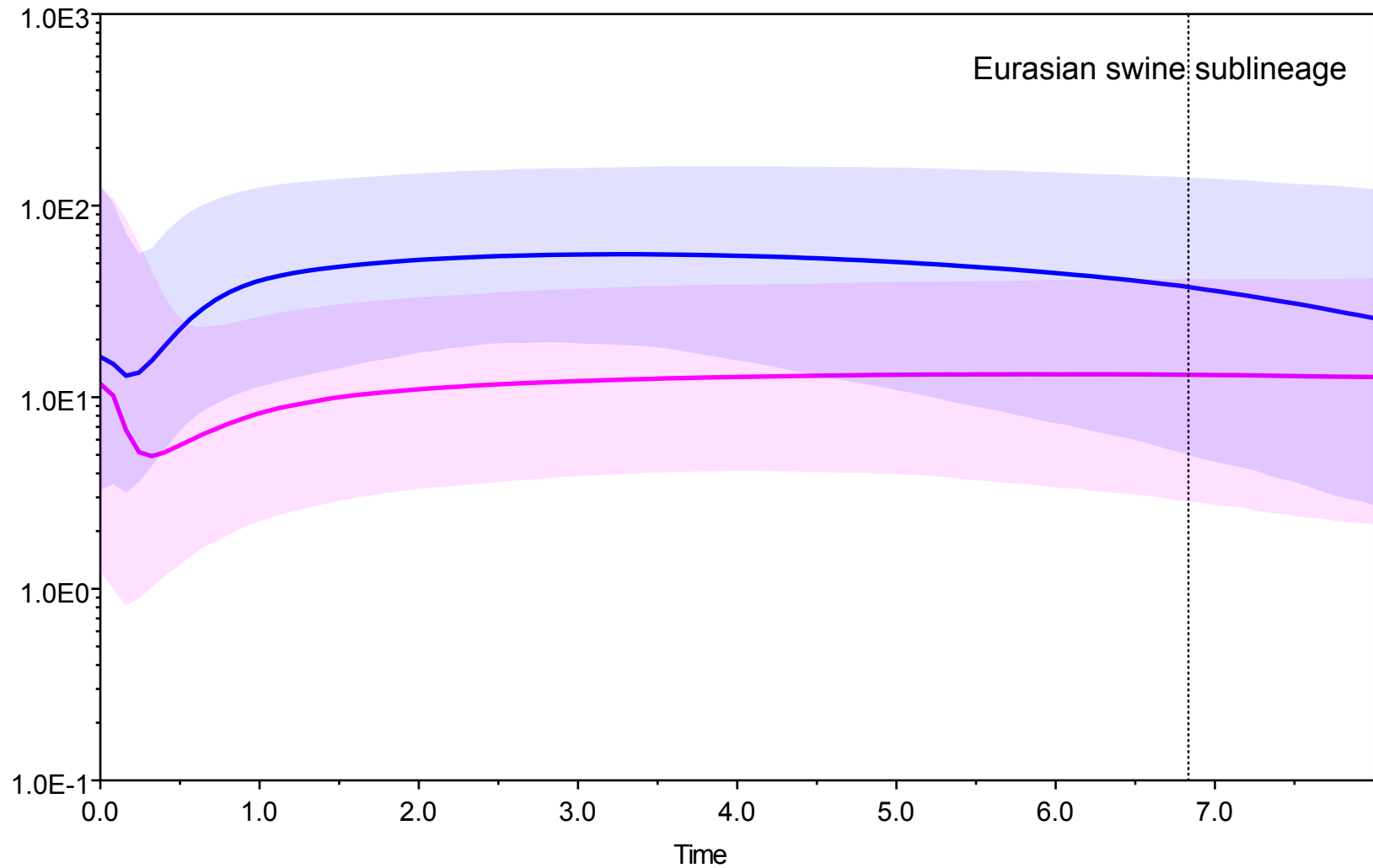

Supplement: Additional file 5 — Figure S3. Population dynamic estimates for classical, Eurasian and avian-to-swine reassortant swine sublineages. X-axis: time in years before present; Y-axis: estimated population size [units = Neτ, the product of effective population size and generation length in years (log-transformed)]. The median estimate and both 95% HPD limits are indicated. [file 1743-422X-8-250-S5.pdf]
