# Supplementary material for: Evolutionary genomics of the pandemic 2009 H1N1 influenza viruses (pH1N 1v)
Source: Virol J. 2011 May 21;8:250. doi: 10.1186/1743-422X-8-250 (PMC3201028; doi:10.1186/1743-422X-8-250)
Supplement: Additional file 3 — Table S1. Nucleotide identities (uncorrected P distance) between 2009 H1N1 viruses and their closest related swine viruses. [file 1743-422X-8-250-S3.PDF]

Virus				Nucleotide identity (%)				
	PB2	PB1	PA	HA	NP	NA	M	NS
Human 2009	0.005746	0.000945	0.000765	0.002228	0.001619	0.002117	0.001086	0.001497
North America and Asia								
A/swine/Korea/CAS08/2005(H1N1)	0.01076		0.007756	0.010577	0.005617			0.005172
	(EU798919)		(EU798879)	(EU798779)	(EU798839)			(EU798859)
A/swine/Korea/CAN01/2004(H1N1)	0.010431		0.007756	0.010811	0.005681			0.005282
	(EU798918)		(EU798878)	(EU798778)	(EU798838)			(EU798858)
A/swine/Ohio/24366/2007(H1N1)	0.01104	0.00752	0.009363	0.008462	0.006838			0.006392
	(EU409946)	(EU409945	(EU409947)	(EU409948)	(EU409950)			(EU409952)
A/swine/OH/511445/2007(H1N1)	0.01104	0.00752	0.009338	0.008287	0.006722			0.006041
	(EU604691)	(EU604692)	(EU604693)	(EU604689)	(EU604694)			(EU604696)
A/swine/NC/00573/2005(H1N1)	0.011109	0.006285	0.008619	0.030147	0.005552			0.008192
	(FJ638303)	(FJ638304)	(FJ638305)	(FJ638306)	(FJ638307)			(FJ638310)
A/swine/IL/00685/2005(H1N1)	0.011189	0.006461	0.008619	0.030005	0.00562			0.008274
	(FJ638295)	(FJ638296)	(EU638297)	(FJ638298)	(FJ638299)			(FJ638302)
A/Iowa/CEID23/2005(H1N1)	0.011031	0.007217	0.007684					
	(DQ889682)	(DQ889683)	(DQ889684)					
A/Wisconsin/10/98 (H1N1)		0.004991						
		(AF342823)						
Europe and Asia								
A/swine/Chachoengsao/NIAH587/2005(H1N1)						0.011828	0.003216	
						(AB434330)	(AB434331)	
A/swine/Chonburi/NIAH589/2005(H1						0.01182	0.003216	

N1)						(AB434322)	(AB434323)	
A/Swine/Spain/50047/2003(H1N1)						0.011508	0.007401	
						(CY009894)	(CY009893)	
A/swine/Zhejiang/1/2007(H1N1)						0.011525	0.006403	
						(FJ415611)	(FJ415612)	
swine/Ille et Vilaine/1455/99 (H1N1)						0.009992	0.005982	
						(AJ410882)	()AJ316061	
A/swine/Spain/53207/2004(H1N1)						0.010479	0.008163	
						(CY010582)	(CY010581)	
A/swine/Spain/51915/2003(H1N1)						0.010479	0.008038	
						(CY010574)	(CY010573)	
A/swine/Chonburi/NIAH977/2004(H1						0.011158		
N1)						(AB434314)		
A/swine/Chonburi/NIAH9469/2004(H						0.011449		
1N1)						(AB434306)		
A/swine/Ratchaburi/NIAH550/2003(H						0.011389		
1N1)						(AB434298)		
A/swine/Ratchaburi/NIAH1481/2000(						0.010843		
H1N1)						(AB434290)		
To swine viruses before 1998	0.029144	0.028341	0.029507	0.024746	0.028335		0.027009	0.010208
To swine viruses before 1988	0.086602	0.074493	0.085181		0.053994	0.092917	0.04117	0.075962
