# Supplementary material for: Evolutionary genomics of the pandemic 2009 H1N1 influenza viruses (pH1N 1v)
Source: Virol J. 2011 May 21;8:250. doi: 10.1186/1743-422X-8-250 (PMC3201028; doi:10.1186/1743-422X-8-250)
Supplement: Additional file 4 — Table S2. Time of recent common ancestors (TMRCA) for 2009 H1N1 viruses and their closest related swine viruses. [file 1743-422X-8-250-S4.DOC]

|  | HA | NA | PA | NP | M1 | NS1 | PB1 | PB2 |
| --- | --- | --- | --- | --- | --- | --- | --- | --- |
| 2009 human H1N1 | 0.341  (0.17-0.55) | 0.381  (0.14-0.71) | 0.179  (0.06-0.33) | 0.437  (0.108-0.883) | 0.467  (0.1-1.0) | 0.315  (0.05-0.7) | 0.1  (0.024-0.216) | 0.3  (0.15-0.547) |
| 2009 H1N1 and closest related swine viruses | 13.68 (8.88-18.75) | 13.86 (8.3-21.41) | 11.63  (6.47-17.72) | 17.37  (10.83-24.64) | 12.77 (6.83-20.68) | 9.31  (5.16-15.18) | 11.95  (7.57-17.65) | 11.65  (6.47-17.72) |
